# Impact of Different Durations of Fasting on Intestinal Autophagy and Serum Metabolome in Broiler Chicken

**DOI:** 10.3390/ani11082183

**Published:** 2021-07-23

**Authors:** Youli Wang, Yanwei Xu, Yuqin Wu, Tahir Mahmood, Jing Chen, Xiaorui Guo, Wei Wu, Bo Wang, Yuming Guo, Jianmin Yuan

**Affiliations:** 1 State Key Laboratory of Animal Nutrition, College of Animal Science and Technology, China Agricultural University, Beijing 100193, China; wangylwy@163.com (Y.W.); xu17801115275@163.com (Y.X.); xiaowu333@foxmail.com (Y.W.); chenjing96518@163.com (J.C.); GXRFY0820@163.com (X.G.); cau_wesir@163.com (W.W.); wangbo123@cau.edu.cn (B.W.); guoyum@cau.edu.cn (Y.G.); 2 Adisseo Animal Nutrition, Dubai 00000, United Arab Emirates; tahir472@gmail.com

**Keywords:** fasting, serum metabolome, broiler chicken, autophagy

## Abstract

**Simple Summary:**

Fasting is usually used before metabolizable energy assessment in poultry. Recently, fasting-induced autophagy has been of concern because of the beneficial function of autophagy. In this study, we found that the intestinal autophagy gene Atg7 has a good quadratic fitting with fasting duration. We found that the serum metabolism pathways involved in glycerophospholipid, phenylalanine, GnRH signaling pathways, glycosylphosphatidylinositol anchor biosynthesis, autophagy, and ferroptosis changed with fasting. Furthermore, we found a correlation between intestinal autophagy and serum metabolite PE (18:3(9Z,12Z,15Z)/P-18:0).

**Abstract:**

Fasting-induced autophagy in the intestine is beneficial for body health. This study was designed to explore the relationship between the host metabolism and intestinal autophagy. Broilers were randomly assigned into 48 cages. At 0 (CT), 12 (FH12), 24 (FH24), 36 (FH36), 48(FH48), and 72 h (FH72) before 09:00 a.m. on day 25, eight cages of birds were randomly allotted to each fasting time point using completely random design, and their food was removed. At 09:00 a.m. on day 25, the blood and jejunum were sampled for serum metabolome and autophagy gene analyses, respectively. The results showed that the autophagy gene Atg7 has a good quadratic fit with fasting duration (R^2^ = 0.432, *p* < 0.001). Serum phosphatidylethanolamine (PE) and lyso-PE were decreased in the birds that were fasted for 24 h or longer. Conversely, the serum phosphatidylcholine (PC) and lyso-PC were increased in the birds that were fasted for 36 h or longer. Metabolism pathway analysis showed that the serum glycerophospholipid, phenylalanine, and GnRH signaling pathways were downregulated with the extended fasting duration. The serum metabolites involved in glycosylphosphatidylinositol anchor biosynthesis, autophagy, and ferroptosis were upregulated in all of the fasted groups. Correlation analysis showed that serum PE (18:3(9Z,12Z,15Z)/P-18:0) was a potential biomarker for intestinal autophagy. Our findings provide a potential biomarker related to intestinal autophagy.

## 1. Introduction

Fasting is becoming more popular in the public because of its beneficial effects, such as anti-aging, anti-inflammatory, and body fat loss [1]. Additionally, fasting is also used to empty the gastrointestinal tract before analyzing the energy efficiency of certain feed ingredient in chickens [2,3,4,5]. During fasting, AMP-activated protein kinase is activated and inhibits the mechanistic target of rapamycin in order to suppress cell growth and activate autophagy [6]. Then, activated autophagy scavenges cytoplasmic materials for energy production [7] and regulates gut homeostasis [8,9]. It has been reported that anomalous autophagy is associated with inflammatory bowel diseases [10] and impaired absorptive enterocytes [8]. What is more, activated intestinal autophagy triggered by fasting protects against TNF-induced apoptosis during chronic colitis and improves life span [11,12]. In vitro, it has been reported that the autophagy level varies with fasting durations [13]. However, the autophagy level in the intestine in response to different fasting durations remain unknown.

It has been suggested that 12 to 24 h of fasting causes a 20% or more decrease in the serum glucose, and depletion of hepatic glycogen in humans [14]. Consequently, fatty acids, ketone bodies, and amino acids are used as energy sources. Traditionally, blood sampling after overnight fasting (≥8 h) is used to monitor body metabolism in humans [15,16,17] and chickens alike [18]. However, broiler chickens are fed ad libitum, except for 1 to 4 h of darkness every day [19,20]. As at least 8 h are needed for emptying the gastrointestinal tract of chickens [21], the broiler’s systemic metabolism can be better characterized by a nonfasted serum. Nowadays, the recommendation of non-fasting blood samples for blood profile assessment in humans has been emerged [22]. Previous studies have been reported the influence of 58 to 96 h of fasting on the host metabolism in humans and mice [23,24]. Serum metabolites have been shown to be involved in digestive function [25,26], which means that serum metabolites may reflect some functions of the intestine. What is more, white blood cells have been recommended to monitor autophagic flux [24]. It is unclear whether there is a metabolite in the serum that is highly correlated with the level of intestinal autophagy.

Liquid chromatography strip–quadrupole mass spectrometry (LC–MS) is regarded as the best choice for vast serum metabolite detection [27]. Therefore, in the present work, we aimed to study the impact of different fasting durations on intestinal autophagy and LC–MS-based untargeted serum metabolites. Furthermore, the potential relationship between serum metabolites and intestinal autophagy was explored.

## 2. Materials and Methods

All of the procedures were complied with the Beijing Regulations of Laboratory Animals, and the Laboratory Animal Ethical Committee of China Agricultural University approved this study (no. AW04110202-3).

### 2.1. Bird Management

A total of 240 one-day-old male broiler chickens (Arbor Acres Plus) were obtained from a commercial hatchery and were randomly assigned to cages with five birds in each cage. The broiler chickens were raised on wire net floors and received a lighting program 23L:1D (turn off at 23:00 p.m. and turn on at 24:00 p.m.) on arrival, which was transformed to a new lighting program 20L:4D (turn off at 22:00 p.m. and turn on at 02:00 a.m.) from day 8. The room temperature was initially set at 33 °C and then gradually decreased according to the age of the birds, until reaching 23 °C on day 21. All of the broilers had free access to water and feed. Broiler chickens were fed a standard corn–soybean meal-based diet, as shown in Table 1. Titanium dioxide (5 g per kg diet) added in the feed to collect digestibility data for other work.

### 2.2. Sample Collection

**Figure 1:**
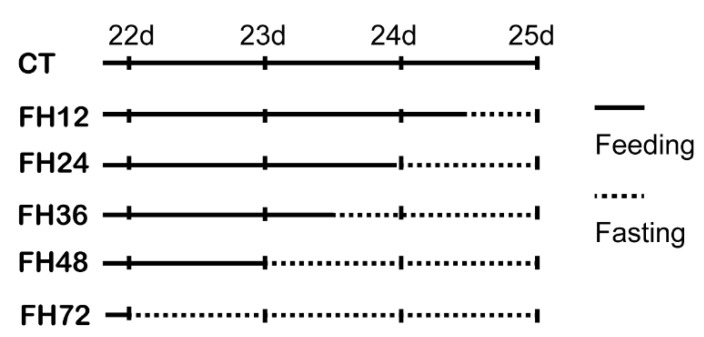
Fasting procedure. CT—birds fed ad libitum; FH12—birds fasted for 12 h; FH24—birds fasted for 24 h; FH36—birds fasted for 36 h; FH48—birds fasted for 48 h.

On day 21, several cages of birds were selected and weighed to get 48 birds with similar body weights. Then, these 48 birds were randomly divided into 48 cages with one bird per cage. At 0 (CT), 12 (FH12), 24 (FH24), 36 (FH36), 48(FH48), and 72 h (FH72) before 09:00 a.m. on day 25, eight cages of birds were randomly allotted to each fasting time point using completely random design, and the food was removed (Figure 1). At 09:00 a.m. on day 25, the blood samples were drawn from all of these birds’ wing veins. The birds were then euthanized by intravenous injection of sodium pentobarbitone (30 mg/kg). Middle jejunum (about 0.25 cm) was collected and washed with saline solution, then snap-frozen in liquid nitrogen immediately, and stored at −80 °C for mRNA analysis. After 4 h of routine temperature shelving, the serum was obtained by centrifugation at 3000 g for 10 min at 4 °C. Samples of jejunum and serum were stored at −80 °C for later analysis.

### 2.3. Chemical Analysis

The feed samples were finely ground and passed through a 40 μm strainer. The gross energy in the feed was determined using an oxygen bomb calorimeter (PARR 6400; Parr Instrument Company, Moline, IL, USA) with benzoic acid as the calibration standard. The nitrogen in the feed was determined using the Kjeldahl method (FOSS KT 200 Kjeltec, Hillerod, Denmark) [28], and CP was calculated using 6.25-fold of nitrogen. The crude fat in the feed was determined in double 1 g samples, packaged into filter-bags (Ankom XT4; ANKOM Technology, Macedon, NY, USA) and extracted by petroleum ether at least 50 times, using a Soxhlet extractor for the ether extract analysis. The starch in the feed was analyzed using a total starch assay kit according to the manufacturer’s instruction (KSTA; Megazyme, Bray, Wicklow, Ireland). Three technical replicate samples were used for the determination of the total calcium and total phosphorus content of the feed using the permanganate titration method and phosphovanoclonoly beate molecular absorption spectrometric method, respectively.

### 2.4. Autophagy-Related Gene Expression

The total RNA was extracted from the chicken jejunum using RNAiso Plus (9109; TAKARA, Kyoto, Japan). The purity and concentration of the total RNA were measured with a nucleic acid analyzer (Nano-drop 2000, Thermo Fisher Scientific, Waltham, MA, USA) using the 260:280 nm absorbance ratio. The complementary DNAs of chicken Atg7 (Accession no. NM_001030592.1) were amplified with the primer as follows: Atg7 sense, CACTGCGGAACTTCCTGATCTTGG, and Atg7 antisense, CTTGCATGGTCCT GTCTCTGAAGC. According to the manufacturer’s instructions, cDNA synthesis was performed using a PrimeScript RT reagent kit with a gDNA eraser. Real-time PCR was conducted on a 7500-fluorescence detection system (Applied Biosystems, Carlsbad, USA) and set as described previously [29]. The mRNA level of Atg7 was normalized against the housekeeping gene β-actin (accession no. XM_027015741.1, amplified as sense CAACACAGTGCTGTCTGGTGGTAC and antisense CTCCTGCTTGCTGATC CACATCTG) using the 2^−ΔΔCt^ method.

### 2.5. Blood Serum Preparation

The blood serum samples stored at −80 °C were thawed at room temperature. Then, the samples were analyzed using LC–MS by OE Biotech. Co., Ltd., in Shanghai. Then, 100 μL of each sample was added to a 1.5 mL tube with 10 μL of 2-chloro-l-phenylalanine (0.3 mg/mL) dissolved in methanol as the internal standard, and then vor-texed for 10 s. Subsequently, 300 μL of the ice-cold mixture of methanol and acetonitrile (2/1, *v*/*v*) was added, and the mixtures were vortexed for 1 min, ultrasonicated at an ambient temperature (25 °C to 28 °C) for 10 min, and stored at −20 °C for 30 min. The extract was centrifuged at 13,000 rpm, 4 °C for 15 min. The supernatants (200 μL) from each tube were collected using crystal syringes, filtered through 0.22 μm micro-filters, and transferred to LC vials. The vials were stored at −80 °C until the LC–MS analysis. Quality control samples were prepared by mixing aliquots of all of the samples to be a pooled sample.

### 2.6. Liquid Chromatography–Mass Spectrometry Analysis

A Shimadzu 30AD UHPLC system fitted with a Q-Exactive quadrupole-Orbitrap mass spectrometer equipped with a heated electrospray ionization source (Thermo Fisher Scientific, Waltham, MA, USA) was used to analyze the metabolic profiling in both the ESI positive and ESI negative ion modes. An ACQUITY UPLC BEH C18 column (1.7 μm, 2.1 × 100 mm) was employed in both the positive and negative modes. The binary gradient elution system consisted of (A) water (containing 0.1% formic acid, *v*/*v*) and (B) acetonitrile (containing 0.1% formic acid, *v*/*v*), and separation was achieved using the following gradients: 5% B over 0–2 min, 20% B over 2–24 min, 100% B over 24–28.1 min, and 5% B over 28.1–30 min. The flow rate was 0.2 mL/min and the column temperature was 40 °C. All of the samples were kept at 4 °C during the analysis. The injection volume was 5 μL.

The mass range was from *m*/*z* 66.7 to 1000.5. The resolution was set at 70,000 for the full MS scans and 17,000 for the HCD MS/MS scans. The collision energy was set at 20, 40, and 60 eV. The mass spectrometer was operated as follows: spray voltage, 3000 V (+) and 2500 V (−); sheath gas flow rate, 35 arbitrary units; auxiliary gas flow rate, 10 arbitrary units; and capillary temperature, 320 °C. The QCs were injected at regular intervals (every 10 samples) throughout the analytical run in order to provide a set of data from which the repeatability could be assessed.

### 2.7. Data Preprocessing and Statistical Analysis

The acquired LC–MS raw data were analyzed by the progenesis QI software (Waters Corporation, Milford, NE, USA) using the following parameters. The precursor tolerance was set to 5 ppm, the fragment tolerance was set to 10 ppm, and the retention time (RT) tolerance was set to 0.02 min. The internal standard detection parameters were deselected for the peak RT alignment, isotopic peaks were excluded for analysis, the noise elimination level was set at 10.00, and the minimum intensity was set to 15% of base peak intensity. The resulting matrix was obtained with three-dimension data sets, including *m*/*z*, peak RT, and peak intensities, and the RT–*m*/*z* pairs were used as the identifier for each ion. The resulting matrix was further reduced by removing any peaks with a missing value (ion intensity = 0) in more than 50% of the samples. The internal standard was used for data quality control.

Metabolites were identified by progenesis QI (Waters Corporation, Milford, NE, USA) Data Processing Software, based on databases such as http://www.hmdb.ca/; http://www.lipidmaps.org/ and self-built databases (accessed on 6 December 2018). The positive and negative data were combined to get combine data that was imported into the R ropes package. Principle component analysis (PCA) and orthogonal partial least-squares-discriminant analysis (OPLS-DA) were carried out to visualize the metabolic alterations among the experimental groups, after mean centering (Ctr) and Pareto variance (Par) scaling, respectively. The Hotelling’s T2 region, shown as an ellipse in the score plots of the models, defines the 95% confidence interval of the modeled variation. Variable importance in the projection (VIP) ranked the overall contribution of each variable to the OPLS-DA model, and those variables with VIP >1 were considered relevant for group discrimination.

In this study, the default seven-round cross-validation was applied, with one/seventh of the samples being excluded from the mathematical model in each round, in order to guard against overfitting. The differential metabolites were selected based on the combination of a statistically significant threshold of variable influence on the projection values obtained from the OPLS-DA model and *p* values from a two-tailed Student’s *t*-test on the normalized peak areas, where metabolites with VIP values larger than 1.0, *p* values less than 0.05, and fold change over 2 were considered as differential metabolites. MBRole was used for the metabolite pathway enrichment analysis.

The data of the Atg7 gene expression were analyzed by ANOVA and Tukey was used in the post hoc test. Pearson’s correlation was used to analyze the correlation between PE and autophagy gene Atg7 using an R statistical package named Corrplot. The values were means, with SEM represented by vertical bars. Treatments with different letters were significantly different (*p* ≤ 0.05).

## 3. Results

### 3.1. Intestinal Autophagy at Different Durations of Fasting

**Figure 2:**
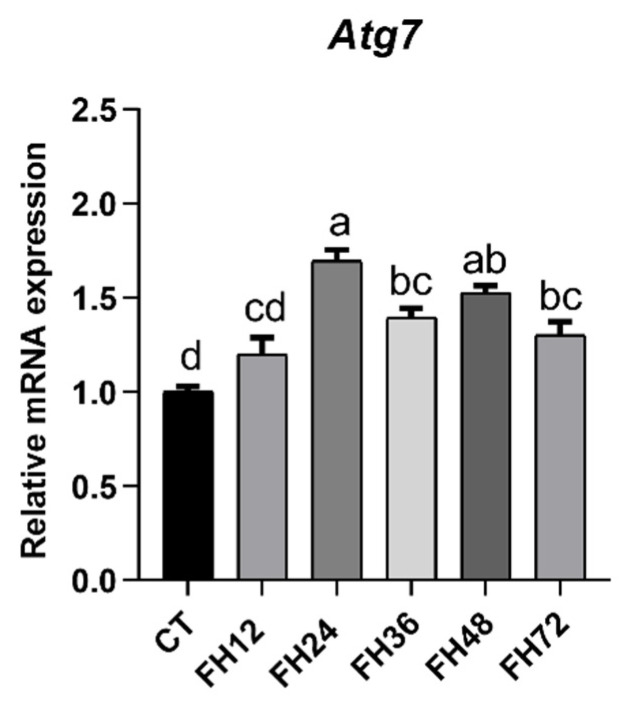
Relative expression of autophagy gene Atg7 at different fasting durations. Data are analyzed by ANOVA and multiple comparisons using the Duncan correction. Values are means ± SEM, *n* = 8. Labeled means without a common letter are significantly different, *p ≤* 0.05. CT—birds fed ad libitum; FH12—birds fasted for 12 h; FH24—birds fasted for 24 h; FH36—birds fasted for 36 h; FH48—birds fasted for 48 h.

This study showed that the mRNA level of Atg 7 was significantly (*p* < 0.05) upregulated at every fasting time point, except for 12 h of fasting (Figure 2). Linear regression did not provide a good fit between the Atg 7 and fasting durations (R^2^ = 0.092, *p =* 0.036), but quadratic fitting provided a good fit (R^2^ = 0.432, *p* < 0.001).

### 3.2. PCA and OPLS-DA Analysis of Chicken Serum at Different Durations of Fasting

**Figure 3:**
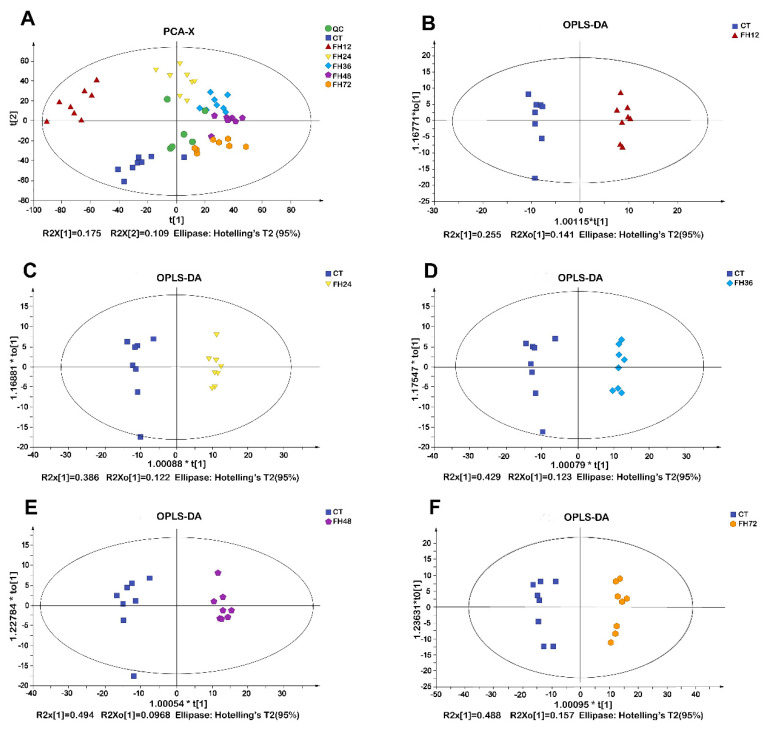
(**A**)Principal component analysis (PCA) and (**B**–**F**) score plots of orthogonal partial least squares discrimination analysis (OPLS-DA) obtained from the non-fasting and fasting groups. CT—birds fed ad libitum; FH12—birds fasted for 12 h; FH24—birds fasted for 24 h; FH36—birds fasted for 36 h; FH48—birds fasted for 48 h.

The PCA score plots suggested a distinct separation among the different fasting durations (Figure 3A). All of the samples in the score plots of the samples were within the 95% Hotelling T2 ellipse. The OPLS-DA revealed a clear separation between the non-fasting and fasting groups (Figure 3B–F). The OPLS-DA models indicated that different fasting time points all significantly affected the serum metabolic patterns.

### 3.3. Serum Metabolic Profiles Changes with Different Durations of Fasting

**Figure 4:**
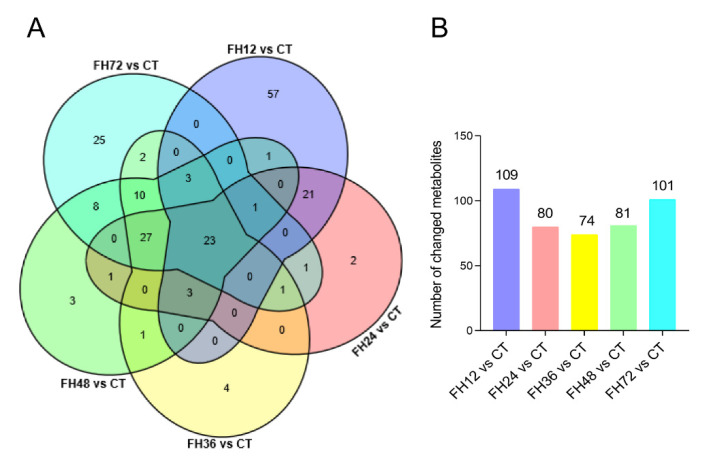
(**A**) Venn diagram of the changed metabolites for different treatments and (**B**) the changed metabolite numbers during different fasting durations when compared with the non-fasting group. CT—birds fed ad libitum; FH12—birds fasted for 12 h; FH24—birds fasted for 24 h; FH36—birds fasted for 36 h; FH48—birds fasted for 48 h.

Compared with the birds in the CT, the number of changed metabolites decreased within 36 h of feed deprivation and increased with longer fasting durations (Figure 4B). The serum metabolomics showed the union set of the changed metabolites between different fasting groups and the non-fasting group reached 194 species, the commonly changed species were 23 species (Figure 4A and Table S1). The metabolites in the union set were fatty acyl groups (52 species, accounting for 26.8%), glycerophospholipids (62 species, accounting for 32.0%), carboxylic acids and derivatives (13 species, 6.7%), steroids and derivatives (7 species, 3.6%), organic oxygen compounds (7 species, 3.6%), organic sulfuric acid and derivatives (6 species, 3.1%), prenol lipids (6 species, 3.1%), indoles and derivatives (4 species, 2.1%), flavonoids (4 species, 2.1%), and other substances (37 species, 19.1%, Table S1).

**Figure 5:**
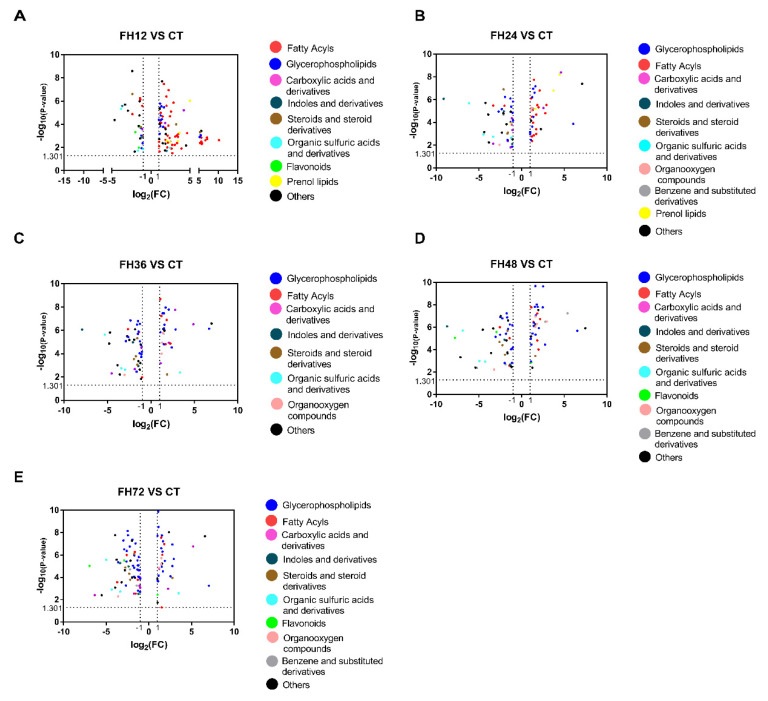
Volcano plots changed at 12 (**A**), 24 (**B**), 36 (**C**), 48 (**D**), and 72 h (**E**) compared with the non-fasting group.

The volcano plots showed that fatty acyls were the most changed metabolites in the birds fasted for 12 h and glycerophospholipids were the most changed metabolites in the extended fasting (Figure 5). Table S2 shows that 90% of the fatty acyl substances were significantly changed in the birds fasted for 12 h, of which 91.5% were increased and 8.5% were significantly decreased. This effect was gradually lost as the fasting time extended.

### 3.4. Changes in Serum Fatty Acyl Metabolism with Different Durations of Fasting

**Figure 6:**
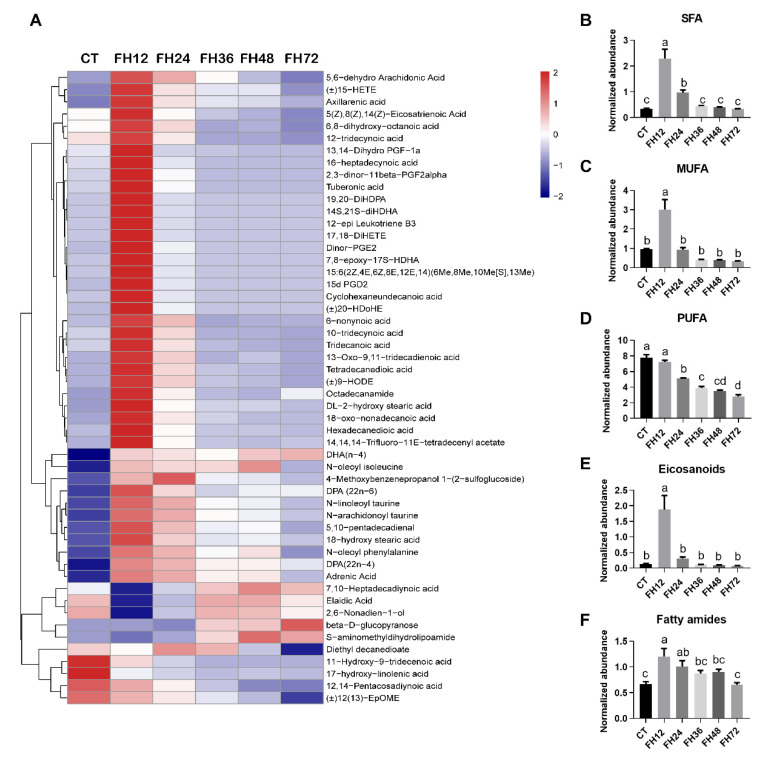
Effect of fasting duration on the serum fatty acyls metabolome. (**A**) Heatmap of fatty acyls during the different fast durations. Changed serum SFA (**B**), MUFA (**C**), PUFA (**D**), eicosanoids (**E**), and fatty amides (**F**) with prolonged fasting duration. Data are analyzed by ANOVA and multiple comparisons using the Duncan correction. Values are means ± SEM, *n* = 8. Labeled means without a common letter are significantly different, *p ≤* 0.05. CT—birds fed ad libitum; FH12—birds fasted for 12 h; FH24—birds fasted for 24 h; FH36—birds fasted for 36 h; FH48—birds fasted for 48 h; SFA—saturated fatty acids; MUFA—monounsaturated fatty acids; PUFA—polyunsaturated fatty acids.

The fatty acyl substances detected in this study mainly consisted of saturated fatty acids (SFA), monoun-saturated fatty acids (MUFA), polyunsaturated fatty acids (PUFA), eicosanoids, and fatty amides (Figure 6A). The level of SFA increased significantly (*p* < 0.05) in FH12 and FH24 and returned to the non-fasting levels in longer fasting durations (Figure 6B). The serum MUFA increased (*p* < 0.05) maximally in FH12, and then decreased to the non-fasting state with prolonged fasting duration (Figure 6C); the serum PUFA level gradually decreased (*p* < 0.05) with the fasting durations extending (Figure 6D). Serum eicosanoid was upregulated in FH12 (*p* < 0.05) and then returned to the non-fasting level in the prolonged fasting durations (Figure 6E and Table S2). It is worth noting that eicosanoids such as the analog of prostaglandin (PG) and leukotriene were significantly increased in the birds of FH12 when compared with the CT (*p* < 0.05, Table S2). The serum lipid amide levels significantly increased (*p* < 0.05) in FH12 and FH24, and then gradually decreased to the non-fasting level in FH72 (Figure 6F).

### 3.5. Changes in Serum Glycerophospholipids Metabolism with Different Durations of Fasting

**Figure 7:**
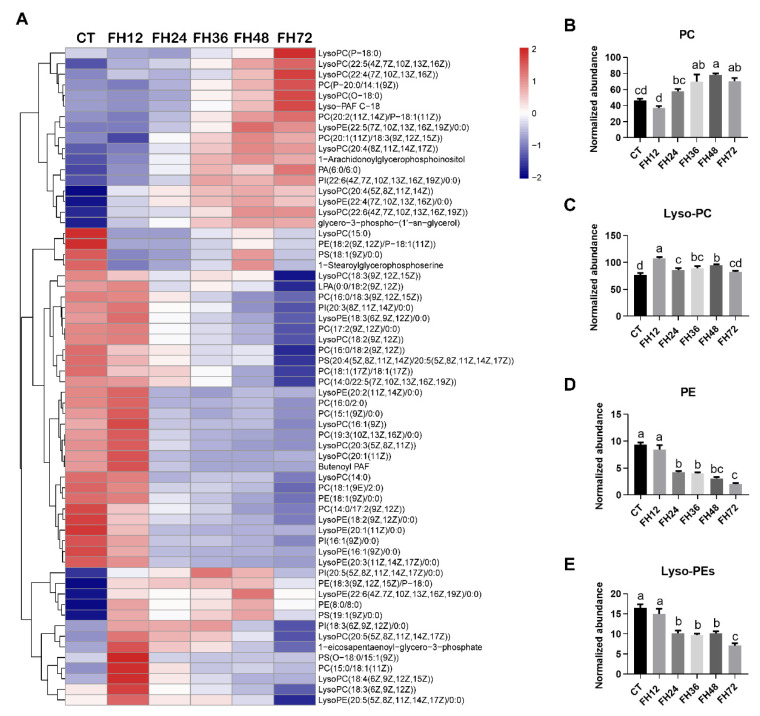
Effect of fasting duration on the serum glycerophospholipid metabolome. (**A**) Heatmap of glycerophospholipids during the different fasting duration. Changes in PC (**B**), Lyso-PC (**C**), PE (**D**), and Lyso-PE (**E**) in response to fasting. Data are analyzed by ANOVA and multiple comparisons using the Duncan correction. Values are means ± SEM, *n* = 8. Labeled means without a common letter are significantly different, *p ≤* 0.05. CT—birds fed ad libitum; FH12—birds fasted for 12 h; FH24—birds fasted for 24 h; FH36—birds fasted for 36 h; FH48—birds fasted for 48 h.

As shown in Figure 7A, the changed serum glycerophospholipids detected in this study were mainly phosphatidylcholine (PC), phosphatidylethanolamine (PE), and their lyso-forms (Lyso-PC and Lyso-PE). The serum PC levels gradually increased with extended fasting durations (*p* < 0.05, Figure 7B). The serum Lyso-PC increased with prolonged fasting durations, except in FH72 (Figure 7C); both the serum PE and Lyso-PE levels gradually decreased with the fasting durations extending (all *p* < 0.05, Figure 7D,E).

### 3.6. Changes in Serum Metabolic Pathway with Different Durations of Fasting

**Figure 8:**
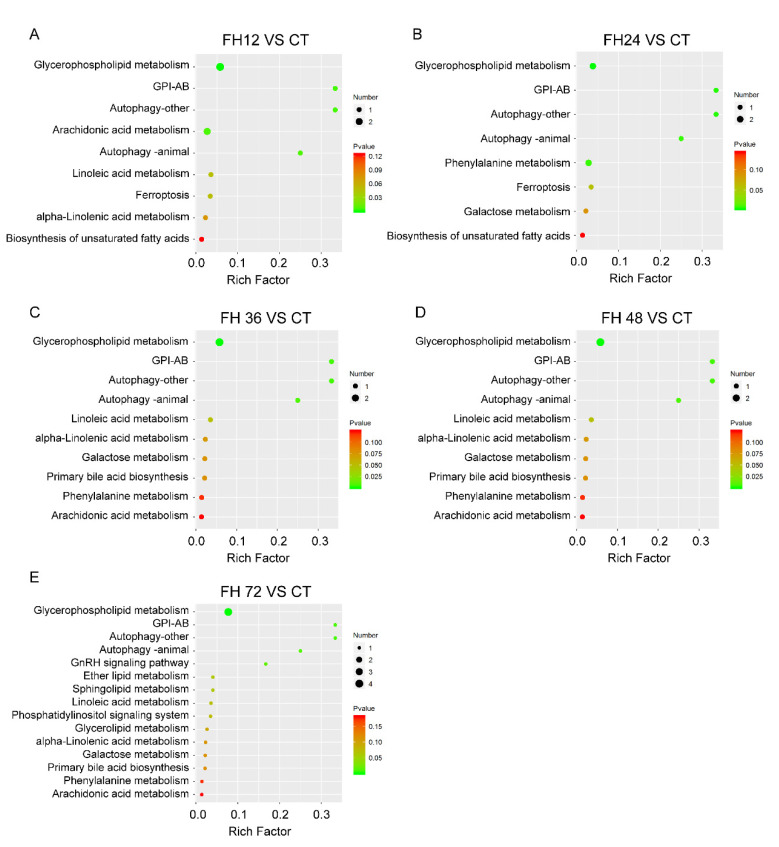
Changed pathways detected in the birds fasted for 12 h (**A**), 24 h (**B**), 36 h (**C**), 48 h (**D**), and 72 h (**E**) when compared with the birds fed ad libitum. CT—birds fed ad libitum; FH12—birds fasted for 12 h; FH24—birds fasted for 24 h; FH36—birds fasted for 36 h; FH48—birds fasted for 48 h.

Through the MBRole pathway enrichment analysis, the serum glycerophospholipid metabolism, glycosylphosphatidylinositol anchor biosynthesis (GPI-AB), autophagy, linoleic acid (LA), arachidonic acid (ADA), phenylalanine metabolism, GnRH signaling pathway, and ferroptosis were identified in the fasted birds when compared with the non-fasted (Figure 8).

**Figure 9:**
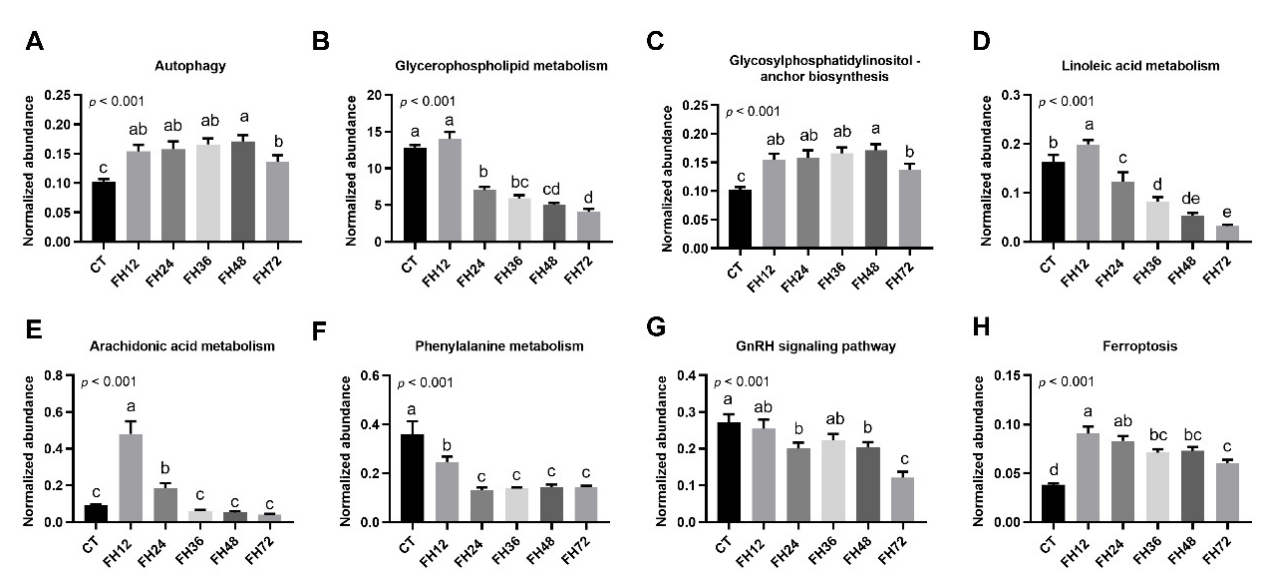
Effects of food withdrawal on the serum metabolome. (**A**–**H**) The normalized abundance of metabolite pathways from the control and fasting groups. Data are analyzed by ANOVA and multiple comparisons using the Duncan correction. Values are means ± SEM, *n* = 8. Labeled means without a common letter are significantly different, *p ≤* 0.05. CT—birds fed ad libitum; FH12—birds fasted for 12 h; FH24—birds fasted for 24 h; FH36—birds fasted for 36 h; FH48—birds fasted for 48 h.

We further evaluated the effect of fasting on the host serum metabolic pathways. The abundance of one metabolic pathway was the sum of the abundance of the detected metabolites typically found in that pathway. The results showed that the abundance of serum autophagy, GPI-AB, and ferroptosis were increased significantly during food deprivation, regardless of fasting durations (all *p* < 0.05, Figure 9A,C,H). The serum glycerophospholipid metabolic pathway was decreased in the birds fasted longer than 24 h (*p* < 0.05, Figure 9B). Both the serum LA and ADA metabolism were increased (all *p* < 0.05) in FH12 and then decreased with prolonged fasting (Figure 9D,E). The abundance of the phenylalanine metabolic pathway was downregulated in all of the fasting groups compared with the non-fasting birds (*p* < 0.05, Figure 9F). The GnRH signaling pathway was gradually decreased with prolonged fasting durations (*p* < 0.05, Figure 9G).

### 3.7. Pearson’s Correlations between Intestinal Autophagy and Serum PE during Fasting

**Figure 10:**
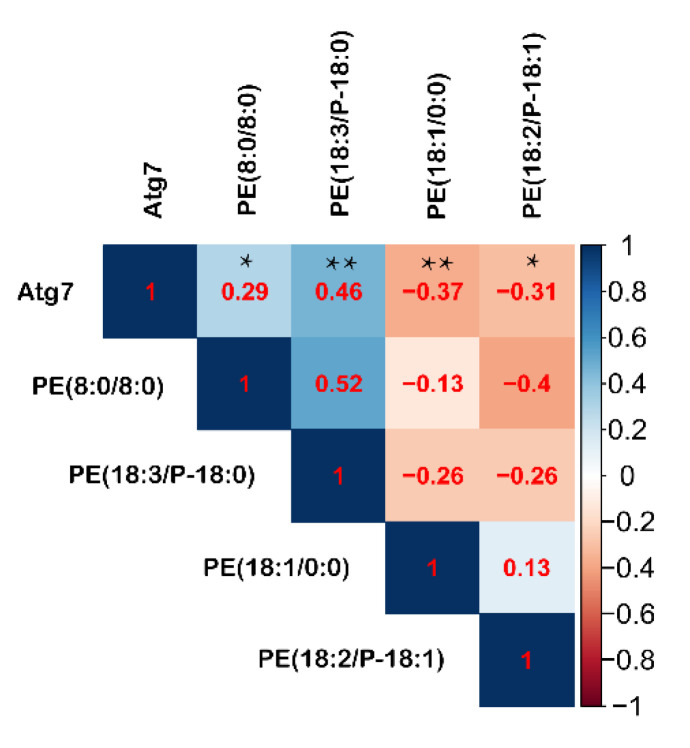
Correlation analysis between intestinal Atg7 and serum PE. Correlation analysis is conducted by the Pearson’s correlation. * means *p* ≤ 0.05, ** means *p* ≤ 0.01.

The serum autophagy pathway showed a quadratic fitting characteristic with fasting durations (R^2^ = 0.380, *p* < 0.001), which was similar to the intestinal autophagy in response to fasting. It implies that the serum autophagy can monitor the intestinal autophagy level. The phosphatidylethanolamine covalent binding to the Atg protein is essential for autophagy initiation, and its abundance positively regulates autophagy. Here, serum metabolome showed that PE (8:0/8:0) and PE (18:3(9Z,12Z,15Z)/P-18:0) were significantly improved during fasting, even fasted for 12 h. Reversely, PE (18:1(9Z)/0:0) and PE (18:2(9Z,12Z)/P-18:1(11Z)) were significantly decreased compared with the control group (Table S2). Correlation analysis showed that PE (18:3(9Z,12Z,15Z)/P-18:0) and PE (8:0/8:0) were positively correlated with Atg7, whereas PE (18:2(9Z,12Z)/P-18:1(11Z)) and PE (18:1(9Z)/0:0) were inversely correlated with Atg7 (Figure 10). According to the correlation coefficient, serum PE (18:3(9Z,12Z,15Z)/P-18:0) is the best biomarker for monitoring intestinal autophagy.

## 4. Discussion

Fasting is widely used in the adjuvant therapy of obesity, inflammation, cardiovascular disease, and so on in humans [1]. In this study, through LC–MS, we identified some altered metabolic pathways at different fasting time points and a potential biomarker in the serum that can predict the intestinal autophagy in chicken.

Autophagy plays an important role in the maintenance of intestinal homeostasis [30]. Generally, autophagy is maintained at a fundamental level in different tissues [31]. Nutritional stress-induced elevated autophagy will harvest the nutrients or damaged organelles for cell survival [32]. Previous work has shown that adult laying hens subjected to 20 days of fasting exhibit large lysosomal autophagic vacuoles in the intestinal epithelial cells and atrophic intestinal villus [33]. It is widely known that Atg7 is a critical autophagy protein that plays an essential role in intestinal integrity [34]. In this study, the genetic expression of Atg7 showed a quadratic response to extended fasting duration, even though previous data indicated an increase in autophagy upon nutrient deprivation [35,36]. Our results showed that 12 h fasting is enough to activate autophagy, and 24 h of fasting had the highest autophagy process. The reverse shift from its peak could potentially point towards a new energy balance inside the body, because longer fasting reduces heat production in the chicken [37]. Intriguingly, a similar pattern in autophagy was also detected in the serum. Increased autophagy is an efficient metabolic process to alleviate inflammation and cell apoptosis [11,38]. It has been reported that fasting reduced inflammatory bowel disease by modulating the microbiota [39]. So, the increased autophagy may provide new evidence for the inflammation pathology. More so, blood cells are a suitable indicator for monitoring autophagic flux [24]. Given that PE covalently binds to the Atg protein during autophagy [40], correlation analysis was applied to the serum PE and intestinal Atg7. We found serum PE (18:3(9Z,12Z,15Z)/P-18:0) is a potential biomarker for intestinal autophagy assessment.

Serum glycerophospholipid was the most detected chemical in the present study. However, the glycerophospholipid metabolism was decreased in the birds fasted for more than 24 h, which is consistent with the results from Maity et al., who reported that starvation decreased glycerophospholipid metabolism in Diporeia [41]. Glycerophospholipid consists of PE, PC, Lyso-PE, and Lyso-PC. It has been reported that Lyso-PC in the plasma is correlated inversely with body mass index [42]. What is more, people refed after food deprivation showed decreased PC and Lyso-PC [43]. Similarly, in the present study, PC and Lyso-PC were increased with fasting. These published data are in agreement with our study. Serum PC is the most abundant phospholipid component in lipoprotein, with the highest levels in the high-density lipoprotein fraction [44]. Increased PC here may imply changes in the lipoprotein metabolism. Serum Lyso-PC, derived from PC by the elimination of one fatty acid by phospholipase A2 and the transesterification by lecithin-cholesterol acyltransferase, accounts for 5–20% of the total phospholipid and participates in many cellular processes [45]. Recently, it has been reported that increased Lyso-PC is associated with suppressed homocysteine thiolactonase activity [45]. Homocysteine thiolactonase may protect against the vascular injury caused by elevated homocysteine levels by preventing its oxidation to the harmful compound, homocysteine thiolactone [46]. So, the increase in Lyso-PC in the present study may indicate that fasting may damage chicken vascular by suppressed homocysteine thiolactonase activity.

Phosphatidylethanolamine is the second most abundant phospholipid in mammalian cells [44]. By catalyzing phospholipase A2, PE was broken into Lyso-PE [47]. A study has shown that cells cocultured with Lyso-PE can inhibit serum starvation-induced cell apoptosis [48]. The decreased Lyso-PE at prolonged fasting in this study may partly explain the atrophic intestinal villus during fasting [49]. Phosphatidylethanolamine participates in the synthesis of glycosylphosphatidylinositol-anchored protein [50] and mediates cell ferroptosis [51,52]. Glycosylphosphatidylinositol, a widely distributed glycolipid in eukaryotes, is the membrane protein’s anchor containing enzymes and receptors [53]. Pantetheinase is one of the glycosylphosphatidylinositol-anchored proteins that hydrolyzes pantetheine into cysteamine and pantothenic acid [54], and elevated pantetheinase has been reported in mice fasted for 24 h when compared with non-fasting mice [55]. Pantothenic acid is a substrate in coenzyme A synthesis that is a necessary cofactor in fatty acid oxidation [56]. So, energy supply during energy deficiency may partly be sourced from increased GPI-AB metabolism in the present study. In turn, serum PE negatively related to GPI-AB and ferroptosis may be attributed to more PE flow into organs and tissues during fasting.

Lipids are the primary energy source for most tissues when glycogen is used up [57]. It was observed that previously birds fasted for 24 and 48 h showed a decreased concentration of plasma triglyceride and free fatty acid compared with the non-fasted [58]. It has been reported that food withdrawal caused a decrease in overnight fasting serum fatty acid metabolism, including SFA, MUFA, and PUFA in mice [59]. In this study, if we considered the birds fasted for 12 h as the control group, and the results aligned well with the previous work conducted in mice [60]. Furthermore, our results provide new evidence for recent guidelines that endorse non-fasting profiles for blood sampling [22,60,61].

Fatty acid-related metabolism pathways such as ADA, LA, and GnRH signaling pathways were detected in this study. Linoleic acid, a precursor of ADA [62], was significantly increased in the birds fasted longer than 12 h and linearly decreased in the birds fasted longer than 12 h. Arachidonic acid can be converted to eicosanoids, such as PG and leukotrienes [62]. In the present study, 12 h and 24 h of fasting increased the serum prostaglandin F2, which is similar to previous work conducted in mice [63,64]. However, long-term fasting leads to decreased prostaglandin D2, E2, and F2. This can be explained by the long-term fasting-induced deficiency of insulin [65]. In summary, serum ADA and LA metabolism are fasting time-dependent.

In addition, phenylalanine has been regarded as an energy source during food deprivation [66]. The stable level of phenylalanine in birds fasted for 24 h or longer suggests that the phenylalanine metabolism provides energy within 24 h of fasting.

## 5. Conclusions

The present study shows that extending the fasting duration from 12 to 72 h influences the serum metabolic pathways, such as the glycerophospholipid metabolism, GPI-AB, autophagy, ADA metabolism, LA metabolism, ferroptosis, phenylalanine metabolism, and GnRH signaling pathway. Our results also suggest that serum PE (18:3(9Z,12Z,15Z)/P-18:0) is a potential biomarker for intestinal autophagy monitoring, and 24 h of fasting activates intestinal autophagy well.

## Figures and Tables

**Table 1:** Ingredient composition of experimental diet ^1^ (as-fed basis).

Item	Composition
Ingredient (%)	
Corn	50.70
Soybean meal (44% CP ^2^)	41.60
Soybean oil	3.30
Limestone	1.35
Di-calcium phosphate	1.43
Sodium chloride	0.35
Vitamins premix ^3^	0.03
Mineral premix ^4^	0.20
Phytase	0.02
Choline chloride (60%)	0.20
DL-Methionine (98%)	0.30
Antioxidant	0.02
Titanium dioxide	0.50
Analyzed nutrient content	
Gross energy (Mcal/kg)	4.03
Crude Protein (%)	21.99
Ether extract	6.00
Total starch	39.12
Calcium (%)	1.15
Total phosphorus (%)	0.56

^1^ Diet is a pellet diet. ^2^ CP, crude protein. ^3^ The vitamin premix provides the following per kg of diets, vit amin A (trans-retinyl acetate) 12,500 IU, vitamin D3 2500 IU, vitamin E (DL-α-tocopherol) 20 IU, vitamin K3 2.65 mg, vitamin B1 2.00 mg, vitamin B2 6.00 mg, vitamin B6 6.00 mg, vitamin B12 0.03 mg, biotin 0.03 mg, folic acid 1.25 mg, pantothenic acid 12.00 mg, and nicotinic acid 50.00 mg. ^4^ The trace mineral premix provided the following per kg of diets: Cu (CuSO_4_•5H_2_O), 8.00 mg; Zn (ZnSO_4_), 75.00 mg; Fe (FeSO_4_•H_2_O), 80.00 mg; Mn (MnSO_4_•H_2_O), 100.00 mg; Se (Na_2_SeO_3_), 0.30 mg; I (KI), 0.35 mg; Co (CoSO_4_•7H_2_O), 0.50 mg.

## Data Availability

The data presented in this study are available on request from the corresponding author.

## Supplementary Materials

The following are available online at https://www.mdpi.com/article/10.3390/ani11082183/s1. Table S1: Common changed metabolites in different fasting groups; Table S2: Changed serum metabolite concentration after different fasting duration.
