# Supplementary material for: Impact of Different Durations of Fasting on Intestinal Autophagy and Serum Metabolome in Broiler Chicken
Source: Animals (Basel). 2021 Jul 23;11(8):2183. doi: 10.3390/ani11082183 (PMC8388447; doi:10.3390/ani11082183)
Supplement: Supplementary file 1 [file animals-11-02183-s001.zip › animals-1273523-supplementary.pdf]

Table S1 Common changed metabolites in different fasting groups

Metabolites
(4E,7E,10Z,13E,16E,19E)-docosa-4,7,10,13,16,19-hexaenoic acid
1-(8-[5]-ladderane-octanyl)-2-(8-[3]-ladderane-octanyl)-sn-glycero-3-phospho-(1'-sn-glycerol)
11-Hydroxy-9-tridecenoic acid
3-Hydroxymelatonin
4-ethylphenylsulfate
4-vinylphenol Sulfate
Acrimarine J
Amoxapine
Apigenin 7-sulfate
Captopril
Dihydronaringenin-O-sulphate
Ferulic acid 4-sulfate
Histidinyl-Lysine
LysoPC(20:4(5Z,8Z,11Z,14Z))
LysoPC(22:5(4Z,7Z,10Z,13Z,16Z))
LysoPC(22:6(4Z,7Z,10Z,13Z,16Z,19Z))
LysoPE(22:4(7Z,10Z,13Z,16Z)/0:0)
LysoPE(22:6(4Z,7Z,10Z,13Z,16Z,19Z)/0:0)
Naringenin 4'-O-glucuronide
N-linoleoyl taurine
Oleoyl Serotonin
PE(18:3(9Z,12Z,15Z)/P-18:0)
Tauroursodeoxycholic acid

Table S2 Changed serum metabolites concentration after different fasting duration

Class	Sub class	Matabolites	Abundance					
			CT	FH12	FH24	FH36	FH48	FH72
Fatty Acyls	Saturated fatty acid	Tridecanoic acid	0.0094	0.0414	0.0179	0.0066	0.0064	0.0051
Fatty Acyls	Saturated fatty acid	18-oxo-nonadecanoic acid	0.0063	0.0692	0.0285	0.0146	0.0102	0.0094
Fatty Acyls	Saturated fatty acid	6,8-dihydroxy-octanoic acid	0.0332	0.0689	0.0405	0.0184	0.0200	0.0129
Fatty Acyls	Saturated fatty acid	Tetradecanedioic acid	0.0395	0.2065	0.1008	0.0458	0.0432	0.0328
Fatty Acyls	Saturated fatty acid	Hexadecanedioic acid	0.1928	1.9638	0.6403	0.2552	0.2600	0.2165
Fatty Acyls	Saturated fatty acid	DL-2-hydroxy stearic acid	0.0620	0.3351	0.1434	0.1041	0.1076	0.0888
Fatty Acyls	Polyunsaturated fatty acid	13-Oxo-9,14-tridecadienoic acid	0.0031	0.0319	0.0150	0.0033	0.0049	0.0019
Fatty Acyls	Polyunsaturated fatty acid	15:6(2Z,4E,6Z,8E,12E,14)(6Me,8Me,10Me[S],13Me)	0.0004	0.0611	0.0064	0.0012	0.0004	0.0006
Fatty Acyls	Polyunsaturated fatty acid	7,10-Heptadecadiynoic acid	0.0475	0.0219	0.0436	0.0564	0.0653	0.0580
Fatty Acyls	Polyunsaturated fatty acid	19,20-DiHDPA	0.0014	0.1486	0.0120	0.0035	0.0028	0.0022
Fatty Acyls	Polyunsaturated fatty acid	DPA(22-4)	0.0213	0.1018	0.0920	0.0741	0.0763	0.0651
Fatty Acyls	Polyunsaturated fatty acid	DPA (22n-6)	0.0810	0.2017	0.1543	0.1259	0.1353	0.1350
Fatty Acyls	Polyunsaturated fatty acid	Adrenic Acid	0.0381	0.0903	0.0830	0.0711	0.0727	0.0600
Fatty Acyls	Polyunsaturated fatty acid	Cyclohexaneundecanoic acid	0.1289	1.9397	0.3493	0.1622	0.1472	0.1310
Fatty Acyls	Polyunsaturated fatty acid	12,14-Pentacosadiynoic acid	5.4099	4.3068	3.4115	2.6534	2.0015	1.8616
Fatty Acyls	Polyunsaturated fatty acid	5,6-dehydro Arachidonic Acid	0.0475	0.1499	0.1155	0.0837	0.0591	0.0383
Fatty Acyls	Polyunsaturated fatty acid	4,7,10,13,16,19-DHA	0.2745	0.6964	0.6692	0.6264	0.7207	0.7474
Fatty Acyls	Polyunsaturated fatty acid	5(Z),8(Z),14(Z)-Eicosatrienoic Acid	0.0262	0.0587	0.0264	0.0185	0.0160	0.0100
Fatty Acyls	Polyunsaturated fatty acid	(±)20-HDoHE	0.0003	0.0375	0.0052	0.0010	0.0008	0.0006
Fatty Acyls	Monounsaturated fatty acid	Axillarenic acid	0.0039	0.0289	0.0154	0.0110	0.0105	0.0075
Fatty Acyls	Monounsaturated fatty acid	16-heptadecynoic acid	0.4726	3.0969	0.5236	0.1799	0.1801	0.1513
Fatty Acyls	Monounsaturated fatty acid	11-Hydroxy-9-tridecenoic acid	0.2275	0.1110	0.0568	0.0362	0.0391	0.0376
Fatty Acyls	Monounsaturated fatty acid	Elaidic Acid	0.0845	0.0329	0.0606	0.0879	0.0881	0.0758
Fatty Acyls	Monounsaturated fatty acid	6-nonynoic acid	0.0819	0.2979	0.1897	0.0725	0.0794	0.0794
Fatty Acyls	Monounsaturated fatty acid	12-tridecynoic acid	0.0460	0.0981	0.0415	0.0121	0.0102	0.0060
Fatty Acyls	Monounsaturated fatty acid	10-tridecynoic acid	0.0249	0.1124	0.0532	0.0165	0.0153	0.0111
Fatty Acyls	Docosanoids	7,8-epoxy-17S-HDHA	0.0001	0.1471	0.0157	0.0034	0.0018	0.0016
Fatty Acyls	Docosanoids	14S,21S-diHDHA	0.0045	0.2566	0.0184	0.0035	0.0023	0.0014
Fatty Acyls	Fatty acid esters	Diethyl decanedioate	0.2513	0.2085	0.3095	0.2743	0.1572	0.0192
Fatty Acyls	Fatty acyl glycosides	4-Methoxybenzenepropanol 1-(2-sulfoglucoside)	0.0159	0.0446	0.0567	0.0329	0.0318	0.0269
Fatty Acyls	Fatty acyl glycosides	beta-D-glucopyranose	0.1841	0.1866	0.1581	0.3669	0.3729	0.5214
Fatty Acyls	Fatty alcohols	2,6-Nonadien-1-ol	0.0463	0.0175	0.0327	0.0455	0.0447	0.0386
Fatty Acyls	Fatty aldehydes	5,10-pentadecadienal	0.0052	0.0606	0.0427	0.0282	0.0292	0.0177
Fatty Acyls	Fatty amides	S-aminomethyldihydrolipoamide	0.0287	0.0194	0.0312	0.0689	0.1028	0.0861
Fatty Acyls	Fatty amides	N-linoleoyl taurine	0.0400	0.3483	0.2752	0.1867	0.1803	0.1393
Fatty Acyls	Fatty amides	N-arachidonoyl taurine	0.0125	0.0586	0.0495	0.0373	0.0351	0.0294
Fatty Acyls	Fatty amides	N-oleoyl phenylalanine	0.0234	0.0932	0.0772	0.0595	0.0676	0.0372
Fatty Acyls	Fatty amides	N-oleoyl isoleucine	0.0464	0.1619	0.1520	0.1490	0.1830	0.1009

Fatty Acyls	Fatty amides	(±)12(13)-EpOME	0.4460	0.3859	0.3374	0.2981	0.2686	0.1815
Fatty Acyls	Fatty amides	Octadecanamide	0.0651	0.1341	0.0874	0.0721	0.0697	0.0815
Fatty Acyls	Fatty esters	14,14,14-Trifluoro-11E-tetradecenyl acetate	0.0011	0.0269	0.0078	0.0023	0.0023	0.0026
Fatty Acyls	Octadecanoids	Tuberonic acid	0.0179	0.1188	0.0308	0.0118	0.0097	0.0093
Fatty Acyls	Octadecanoids	17-hydroxy-linolenic acid	0.0402	0.0078	0.0040	0.0024	0.0030	0.0022
Fatty Acyls	Octadecanoids	18-hydroxy stearic acid	0.0550	0.2166	0.1500	0.1166	0.1221	0.1021
Fatty Acyls	Lineolic acids and derivatives	(±)9-HODE	0.0529	0.2238	0.1250	0.0658	0.0563	0.0455
Fatty Acyls	Eicosanoids	Dinor-PGE2	0.0008	0.0314	0.0058	0.0013	0.0013	0.0011
Fatty Acyls	Eicosanoids	2,3-dinor-11beta-PGF2alpha	0.0899	0.7496	0.1686	0.0516	0.0454	0.0364
Fatty Acyls	Eicosanoids	15d PGD2	0.0113	0.5736	0.0849	0.0281	0.0244	0.0199
Fatty Acyls	Eicosanoids	17,18-DiHETE	0.0052	0.2614	0.0169	0.0038	0.0025	0.0011
Fatty Acyls	Eicosanoids	13,14-Dihydro PGF-1a	0.0264	0.1635	0.0182	0.0090	0.0080	0.0072
Fatty Acyls	Eicosanoids	12-epi Leukotriene B3	0.0012	0.0675	0.0040	0.0010	0.0005	0.0003
Fatty Acyls	Eicosanoids	(±)15-HETE	0.0053	0.0314	0.0171	0.0124	0.0122	0.0060
Glycerophospholipids	Glycerophosphocholines	PC(16:0/2:0)	0.4843	0.5436	0.1971	0.1967	0.2056	0.1486
Glycerophospholipids	Glycerophosphocholines	PC(15:0/18:1(11Z))	0.0010	0.0384	0.0159	0.0067	0.0072	0.0050
Glycerophospholipids	Glycerophosphocholines	PC(15:1(9Z)/0:0)	0.1205	0.1451	0.0599	0.0501	0.0568	0.0412
Glycerophospholipids	Glycerophosphocholines	PC(18:1(17Z)/18:1(17Z))	16.6429	13.8711	13.8087	11.9087	9.8205	6.9538
Glycerophospholipids	Glycerophosphocholines	PC(18:1(9E)/2:0)	0.7671	0.7094	0.4555	0.4636	0.4246	0.3212
Glycerophospholipids	Glycerophosphocholines	PC(P-20:0/14:1(9Z))	0.0494	0.0339	0.0736	0.1393	0.2012	0.2868
Glycerophospholipids	Glycerophosphocholines	PC(14:0/17:2(9Z,12Z))	0.0680	0.0437	0.0292	0.0240	0.0143	0.0092
Glycerophospholipids	Glycerophosphocholines	PC(14:0/22:5(7Z,10Z,13Z,16Z,19Z))	2.3469	2.3051	2.0354	1.8337	1.5271	1.1541
Glycerophospholipids	Glycerophosphocholines	PC(16:0/18:2(9Z,12Z))	0.5211	0.4177	0.3841	0.3297	0.3393	0.1610
Glycerophospholipids	Glycerophosphocholines	PC(16:0/18:3(9Z,12Z,15Z))	0.1619	0.1604	0.1068	0.0748	0.0459	0.0269
Glycerophospholipids	Glycerophosphocholines	PC(17:2(9Z,12Z)/0:0)	0.2698	0.2749	0.1979	0.1820	0.1635	0.1170
Glycerophospholipids	Glycerophosphocholines	PC(19:3(10Z,13Z,16Z)/0:0)	0.0589	0.0740	0.0257	0.0194	0.0164	0.0131
Glycerophospholipids	Glycerophosphocholines	PC(20:1(11Z)/18:3(9Z,12Z,15Z))	27.5146	18.0630	43.7408	54.4455	67.0540	60.9672
Glycerophospholipids	Glycerophosphocholines	PC(20:2(11Z,14Z)/P-18:1(11Z))	0.0104	0.0059	0.0200	0.0387	0.0599	0.0706
Glycerophospholipids	Glycerophosphocholines	LysoPC(14:0)	0.9985	0.9500	0.5028	0.5466	0.5411	0.4115
Glycerophospholipids	Glycerophosphocholines	LysoPC(15:0)	0.0623	0.0267	0.0267	0.0335	0.0390	0.0323
Glycerophospholipids	Glycerophosphocholines	LysoPC(O-18:0)	0.1952	0.1894	0.2047	0.2750	0.3147	0.4152
Glycerophospholipids	Glycerophosphocholines	LysoPC(P-18:0)	0.0532	0.0446	0.0416	0.0561	0.0637	0.1072
Glycerophospholipids	Glycerophosphocholines	LysoPC(16:1(9Z))	9.7593	12.9896	5.7182	5.2958	5.3413	3.8474
Glycerophospholipids	Glycerophosphocholines	LysoPC(18:2(9Z,12Z))	35.6570	36.0840	26.2554	24.5911	21.5943	15.7535
Glycerophospholipids	Glycerophosphocholines	LysoPC(18:3(6Z,9Z,12Z))	2.0171	3.2735	1.8945	1.6714	1.3750	0.8034
Glycerophospholipids	Glycerophosphocholines	LysoPC(18:3(9Z,12Z,15Z))	0.1196	0.1070	0.0924	0.1030	0.0982	0.0575
Glycerophospholipids	Glycerophosphocholines	LysoPC(18:4(6Z,9Z,12Z,15Z))	0.0008	0.0302	0.0101	0.0041	0.0005	0.0000
Glycerophospholipids	Glycerophosphocholines	LysoPC(20:1(11Z))	0.3546	0.4283	0.1904	0.1672	0.1649	0.1749
Glycerophospholipids	Glycerophosphocholines	LysoPC(20:3(5Z,8Z,11Z))	8.2626	9.9720	4.0042	3.0921	2.5756	2.1759
Glycerophospholipids	Glycerophosphocholines	LysoPC(20:4(5Z,8Z,11Z,14Z))	11.2274	25.1516	28.8086	31.5943	33.2249	30.9704
Glycerophospholipids	Glycerophosphocholines	LysoPC(20:4(8Z,11Z,14Z,17Z))	0.9907	1.1725	1.8625	2.5000	3.0368	2.8477
Glycerophospholipids	Glycerophosphocholines	LysoPC(20:5(5Z,8Z,11Z,14Z,17Z))	2.0177	4.8540	4.0495	3.8667	2.8558	1.4127
Glycerophospholipids	Glycerophosphocholines	LysoPC(22:4(7Z,10Z,13Z,16Z))	1.2996	1.8002	1.6140	2.0099	2.8871	4.0731

Glycerophospholipids	Glycerophosphocholines	LysoPC(22:5(4Z,7Z,10Z,13Z,16Z))	1.4758	3.8860	4.5343	6.0916	8.2384	9.9825
Glycerophospholipids	Glycerophosphocholines	LysoPC(22:6(4Z,7Z,10Z,13Z,16Z,19Z))	2.4891	5.7725	5.9374	7.3494	9.2254	9.2175
Glycerophospholipids	Glycerophosphoethanolamines	PE(8:0/8:0)	0.0143	0.0421	0.0344	0.0362	0.0416	0.0329
Glycerophospholipids	Glycerophosphoethanolamines	PE(18:1(9Z)/0:0)	8.6887	8.2277	4.3154	3.5879	2.9036	1.8358
Glycerophospholipids	Glycerophosphoethanolamines	PE(18:2(9Z,12Z)/P-18:1(11Z))	0.0491	0.0203	0.0198	0.0237	0.0289	0.0235
Glycerophospholipids	Glycerophosphoethanolamines	PE(18:3(9Z,12Z,15Z)/P-18:0)	0.0527	0.1337	0.1382	0.1421	0.1422	0.1128
Glycerophospholipids	Glycerophosphoethanolamines	LysoPE(16:1(9Z)/0:0)	0.7926	0.5304	0.1221	0.1074	0.0961	0.0659
Glycerophospholipids	Glycerophosphoethanolamines	LysoPE(18:2(9Z,12Z)/0:0)	10.7239	6.9947	4.3533	3.8424	3.2108	1.9211
Glycerophospholipids	Glycerophosphoethanolamines	LysoPE(18:3(6Z,9Z,12Z)/0:0)	0.5050	0.5754	0.3246	0.2568	0.1950	0.0931
Glycerophospholipids	Glycerophosphoethanolamines	LysoPE(20:1(11Z)/0:0)	0.0811	0.0471	0.0163	0.0132	0.0142	0.0132
Glycerophospholipids	Glycerophosphoethanolamines	LysoPE(20:2(11Z,14Z)/0:0)	0.4087	0.4541	0.2088	0.1866	0.2158	0.2136
Glycerophospholipids	Glycerophosphoethanolamines	LysoPE(20:3(11Z,14Z,17Z)/0:0)	1.3755	1.1062	0.3582	0.2683	0.2459	0.1753
Glycerophospholipids	Glycerophosphoethanolamines	LysoPE(20:5(5Z,8Z,11Z,14Z,17Z)/0:0)	0.5393	0.7595	0.5494	0.5708	0.4688	0.2318
Glycerophospholipids	Glycerophosphoethanolamines	LysoPE(22:4(7Z,10Z,13Z,16Z)/0:0)	0.0708	0.1656	0.2037	0.2281	0.2446	0.2231
Glycerophospholipids	Glycerophosphoethanolamines	LysoPE(22:5(7Z,10Z,13Z,16Z,19Z)/0:0)	0.0343	0.0344	0.0513	0.0716	0.1093	0.0984
Glycerophospholipids	Glycerophosphoethanolamines	LysoPE(22:6(4Z,7Z,10Z,13Z,16Z,19Z)/0:0)	1.9582	4.3011	3.9676	4.1704	5.3221	4.0351
Glycerophospholipids	Glycerophosphates	PA(6:0/6:0)	0.0008	0.0264	0.0556	0.0857	0.0767	0.1088
Glycerophospholipids	Glycerophosphates	LPA(0:0/18:2(9Z,12Z))	0.2710	0.2850	0.2002	0.2256	0.2054	0.1211
Glycerophospholipids	Glycerophosphates	1-eicosapentaenoyl)-glycero-3-phosphate	0.0696	0.2211	0.1497	0.1325	0.0844	0.0336
Glycerophospholipids	Glycerophosphoinositols	PI(16:1(9Z)/0:0)	0.0826	0.0618	0.0266	0.0264	0.0210	0.0129
Glycerophospholipids	Glycerophosphoinositols	PI(18:3(6Z,9Z,12Z)/0:0)	0.0277	0.0600	0.0581	0.0617	0.0339	0.0178
Glycerophospholipids	Glycerophosphoinositols	PI(20:3(8Z,11Z,14Z)/0:0)	0.1981	0.2135	0.1400	0.1345	0.0912	0.0603
Glycerophospholipids	Glycerophosphoinositols	PI(20:5(5Z,8Z,11Z,14Z,17Z)/0:0)	0.0141	0.0453	0.0526	0.0742	0.0617	0.0372
Glycerophospholipids	Glycerophosphoinositols	PI(22:6(4Z,7Z,10Z,13Z,16Z,19Z)/0:0)	0.0104	0.0241	0.0396	0.0744	0.0698	0.0791
Glycerophospholipids	Glycerophosphoserines	PS(18:1(9Z)/0:0)	0.4566	0.1999	0.2148	0.2628	0.3942	0.2546
Glycerophospholipids	Glycerophosphoserines	PS(19:1(9Z)/0:0)	0.2165	0.4532	0.3784	0.4265	0.4649	0.3565
Glycerophospholipids	Glycerophosphoserines	PS(20:4(5Z,8Z,11Z,14Z)/20:5(5Z,8Z,11Z,14Z,17Z))	0.0863	0.0730	0.0608	0.0514	0.0545	0.0240
Glycerophospholipids	Glycerophosphoserines	PS(O-18:0/15:1(9Z))	0.0011	0.0477	0.0015	0.0005	0.0006	0.0005
Glycerophospholipids	Glycerophosphocholines	Butenoyl PAF	0.4231	0.5172	0.2435	0.2036	0.2099	0.2119
Glycerophospholipids	Glycerophosphocholines	Lyso-PAF C-18	0.3046	0.2928	0.3347	0.4124	0.5134	0.6724
Glycerophospholipids	Glycerophosphoglycerols	glycero-3-phospho-(1'-sn-glycerol)	0.1908	0.3829	0.4554	0.5407	0.5947	0.5801
Glycerophospholipids	Glycerophosphoinositols	1-Arachidonoylglycerophosphoinositol	0.3921	0.4662	0.6175	0.7660	0.8321	0.7761
Glycerophospholipids	Glycerophosphoserines	1-Stearoylglycerophosphoserine	0.1218	0.0579	0.0651	0.0763	0.1103	0.0706
Carboxylic acids and derivatives	Amino acids, peptides, and analogues	S-(2-carboxypropyl)-Cysteamine	0.2356	0.1409	0.1321	0.1631	0.1703	0.1110
Carboxylic acids and derivatives	Amino acids, peptides, and analogues	Cystathionine sulfoxide	0.1620	0.0943	0.0161	0.0070	0.0043	0.0020
Carboxylic acids and derivatives	Amino acids, peptides, and analogues	Alpha-N-Phenylacetyl-L-glutamine	0.2206	0.1530	0.0726	0.0609	0.0602	0.0667
Carboxylic acids and derivatives	Amino acids, peptides, and analogues	Captopril	0.0026	0.0475	0.0630	0.0761	0.1077	0.0943
Carboxylic acids and derivatives	Amino acids, peptides, and	L-gamma-glutamyl-L-isoleucine	0.7902	0.3677	0.3545	0.4632	0.5549	0.4644

	analogues							
Carboxylic acids and derivatives	Amino acids, peptides, and analogues	Tyrosyl-Hydroxyproline	0.7240	0.4158	0.3286	0.3380	0.3695	0.3234
Carboxylic acids and derivatives	Amino acids, peptides, and analogues	Tranexamic Acid	0.1579	0.0821	0.0757	0.0971	0.1135	0.0753
Carboxylic acids and derivatives	Amino acids, peptides, and analogues	Capryloylglycine	0.0432	0.0492	0.0806	0.0936	0.0737	0.1007
Carboxylic acids and derivatives	Amino acids, peptides, and analogues	Histidinyl-Lysine	0.0265	0.0935	0.1580	0.1818	0.1843	0.1266
Carboxylic acids and derivatives	Amino acids, peptides, and analogues	L-cysteine Hydrochloride	0.3740	0.4579	0.4443	0.3100	0.2544	0.1837
Carboxylic acids and derivatives	Amino acids, peptides, and analogues	N-Acetyl-D-phenylalanine	0.1389	0.0920	0.0569	0.0765	0.0831	0.0759
Carboxylic acids and derivatives	Carboxylic acid derivatives	Dihydroceramide	0.2298	0.1390	0.1481	0.1604	0.1463	0.1147
Carboxylic acids and derivatives	Tricarboxylic acids and derivatives	Garcinia lactone dibutyl ester	0.0200	0.0090	0.0507	0.0831	0.0722	0.0626
Steroids and steroid derivatives	Androstane steroids	Androstenol	0.0016	0.0647	0.0210	0.0101	0.0085	0.0062
Steroids and steroid derivatives	Bile acids, alcohols and derivatives	Sulfolithocholylglycine	1.2333	0.7075	0.3887	0.3697	0.2621	0.2505
Steroids and steroid derivatives	Bile acids, alcohols and derivatives	Tauroursodeoxycholic acid	0.2556	0.0493	0.0592	0.0862	0.0815	0.0780
Steroids and steroid derivatives	Bile acids, alcohols and derivatives	Lithocholyltaurine	0.1336	0.0553	0.2218	0.4841	0.4094	0.9043
Steroids and steroid derivatives	Bile acids, alcohols and derivatives	7 alpha-Hydroxy-3-oxo-4-cholestenoate	0.5142	1.4790	0.2899	0.1737	0.0859	0.0761
Steroids and steroid derivatives	Bile acids, alcohols and derivatives	N-oleoyl taurine	0.0037	0.0345	0.0307	0.0248	0.0302	0.0252
Steroids and steroid derivatives	Vitamin D and derivatives	24-Hydroxycalcitriol	0.0805	0.1742	0.0267	0.0206	0.0208	0.0177
Sphingolipids	Phosphosphingolipids	SM(d17:1/17:0)	2.8133	3.0049	3.8691	4.1575	4.3908	5.6315
Sphingolipids	Sphingoid bases	Sphingofungin B	0.0368	0.1381	0.0529	0.0251	0.0207	0.0275
Organic sulfuric acids and derivatives	Arylsulfates	O-methoxycatechol-O-sulphate	0.0079	0.0374	0.0354	0.0800	0.0468	0.0890
Organic sulfuric acids and derivatives	Arylsulfates	Pyrocatechol sulfate	0.8954	0.4451	0.0889	0.1128	0.0462	0.0869
Organic sulfuric acids and derivatives	Arylsulfates	2-methoxyacetaminophen sulfate	0.0647	0.3513	0.2025	0.0418	0.0256	0.0222
Organic sulfuric acids and derivatives	Arylsulfates	4-vinylphenol Sulfate	0.1349	0.0096	0.0019	0.0034	0.0011	0.0042
Organic sulfuric acids and derivatives	Arylsulfates	4-ethylphenylsulfate	0.9046	0.2864	0.0409	0.0847	0.0295	0.0444
Organic sulfuric acids and derivatives	Arylsulfates	Indoxylsulfuric acid	0.3151	0.1557	0.1387	0.3014	0.1900	0.2949
Prenol lipids	Monoterpenoids	(4S,8R)-8,9-Dihydroxy-p-menth-1(6)-en-2-one	0.0728	0.0376	0.0298	0.0190	0.0215	0.0164
Prenol lipids	Sesquiterpenoids	3-Hydroxy-beta-ionone	0.0254	0.1379	0.0798	0.0399	0.0334	0.0351
Prenol lipids	Sesquiterpenoids	Capsidiol	0.0074	0.0812	0.0246	0.0070	0.0064	0.0042
Prenol lipids	Sesquiterpenoids	Germacrenone	0.1059	0.4328	0.1363	0.0454	0.0473	0.0343
Prenol lipids	Sesquiterpenoids	4-(2,6,6-Trimethyl-1-cyclohexen-1-yl)-2-butanone	0.0030	0.0381	0.0401	0.0296	0.0344	0.0248

Prenol lipids	Sesquiterpenoids	7,8-Dehydro-3,4-dihydro-beta-ionol	0.0029	0.0909	0.0626	0.0362	0.0402	0.0315
Organooxygen compounds	Alcohols and polyols	Isokobusone	0.0084	0.0459	0.0100	0.0043	0.0022	0.0026
Organooxygen compounds	Carbohydrates and carbohydrate conjugates	Galactitol	0.2103	0.1264	0.0350	0.0255	0.0227	0.0174
Organooxygen compounds	Carbohydrates and carbohydrate conjugates	(x)-1,2-Propanediol 1-O-b-D-glucopyranoside	0.0375	0.0529	0.0687	0.0885	0.1291	0.1017
Organooxygen compounds	Carbohydrates and carbohydrate conjugates	N-Acetylmuramoyl-Ala	0.0080	0.0142	0.0279	0.0378	0.0621	0.0245
Organooxygen compounds	Carbonyl compounds	2-Thiophenecarboxaldehyde	0.0536	0.0893	0.1183	0.1414	0.1649	0.1488
Organooxygen compounds	Carbonyl compounds	10beta-12,13-Dinor-8-oxo-6-eremophilen-11-al	0.0207	0.0725	0.0452	0.0246	0.0127	0.0097
Organooxygen compounds	Ethers	(2Z,4'Z)-2-(5-Methylthio-4-penten-2-ynylidene)-1,6-dioxaspiro[4.4]non-3-ene	0.0430	0.0562	0.0965	0.1055	0.1329	0.0993
Indoles and derivatives	Indolecarboxylic acids and derivatives	Indole-3-carboxilic acid-O-sulphate	0.0228	0.0918	0.0214	0.0149	0.0059	0.0041
Indoles and derivatives	Indolines	3-Hydroxymelatonin	0.0871	0.0069	0.0002	0.0004	0.0002	0.0085
Indoles and derivatives	Indolyl carboxylic acids and derivatives	Indolelactic acid	0.1346	0.0771	0.0629	0.0491	0.0531	0.0573
Indoles and derivatives	Tryptamines and derivatives	Oleoyl Serotonin	0.1959	0.0886	0.0465	0.0464	0.0492	0.0377
Benzene and substituted derivatives	Benzoic acids and derivatives	Vanilloylglycine	0.2002	0.1999	0.0462	0.0300	0.0202	0.0435
Benzene and substituted derivatives	Halobenzenes	Lofexidine	0.0274	0.0451	0.0704	0.0811	0.1071	0.0885
Benzene and substituted derivatives	Phenylpropanes	Probucol	0.7433	0.2852	0.3268	0.4634	0.3101	0.2780
Dibenzocycloheptenes	Unclassified	Desmethylnortriptyline	0.4736	0.2512	0.1397	0.1948	0.1498	0.0675
Carboximidic acids and derivatives	Carboximidic acids	Hexamethylene bisacetamide	0.0254	0.0261	0.0316	0.0301	0.0261	0.1303
Phenols	1-hydroxy-2-unsubstituted benzenoids	6,7-Dihydro-4-(hydroxymethyl)-2-(p-hydroxyphenethyl)-7-methyl-5H-2-pyrindinium	0.0472	0.0980	0.0500	0.0438	0.0465	0.0483
Phenols	4-alkoxyphenols	4-Hydroxyatomoxetine	0.0114	0.0414	0.0123	0.0197	0.0175	0.0205
Glycerolipids	Diradylglycerols	1-(14-methyl-pentadecanoyl)-2-(8-[3]-ladderane-octanyl)-sn-glycerol	0.1448	0.0487	0.0867	0.1146	0.1436	0.1228
Glycerolipids	Diradylglycerols	DG(22:5(4Z,7Z,10Z,13Z,16Z)/22:6(4Z,7Z,10Z,13Z,16Z,19Z)/0:0)	2.4752	2.3138	1.2039	0.9026	0.7894	0.6369
Hydroxy acids and derivatives	Beta hydroxy acids and derivatives	3-hydroxy-3-(3-hydroxyphenyl)propanoic acid-O-sulphate	0.0265	0.0027	0.0010	0.0009	0.0010	0.0011
Benzopyrans	1-benzopyrans	Artonol E	0.0661	0.0915	0.1258	0.1773	0.2056	0.1919
Benzopyrazoles	Indazoles	Nigellidine	0.1566	0.0784	0.0076	0.0108	0.0011	0.0102
Benzothiophenes	Dibenzothiophenes	4-Methyldibenzothiophene	0.1004	0.0871	0.1232	0.1777	0.2174	0.1905
Benzoxazepines	Dibenzoxazepines	Amoxapine	0.0008	0.0392	0.1067	0.0980	0.1390	0.0744

Dihydrofurans	Furanones	Ascorbic acid-2-sulfate	0.0864	0.1706	0.1879	0.1561	0.2078	0.1264
Naphthofurans	Unclassified	2,3-Secoporrigenin	0.0106	0.0369	0.0508	0.0391	0.0356	0.0124
Naphthopyrans	Unclassified	Janthitrem B	0.0837	0.1811	0.1332	0.1414	0.1116	0.0546
Pyrans	Pyranones and derivatives	Erinapyrone C	0.1006	0.0191	0.0609	0.0972	0.0824	0.0912
Pyrrolidines	N-alkylpyrrolidines	N-(3-acetamidopropyl)pyrrolidin-2-one	0.1501	0.1262	0.1866	0.2435	0.2754	0.3067
Pyrrolopyrazines	Cyclopyrrolones	Eszopiclone	0.0171	0.0459	0.0210	0.0195	0.0195	0.0178
Quinolines and derivatives	Benzoquinolines	Acrimarine J	0.2563	0.0922	0.1065	0.0723	0.0472	0.0166
Quinolines and derivatives	Benzoquinolines	Neoacrimarine A	0.1395	0.1634	0.0921	0.0608	0.0464	0.0355
Tetrahydrofurans	Unclassified	Riesling acetal	0.0120	0.0434	0.0208	0.0055	0.0083	0.0066
Alcohols and polyols	Secondary alcohols	8-Hydroxy-4(6)-lactarene-5,14-diol	0.0333	0.0950	0.0433	0.0268	0.0313	0.0281
Cinnamic acids and derivatives	Hydroxycinnamic acids and derivatives	Ferulic acid 4-sulfate	0.3596	0.0467	0.0192	0.0137	0.0153	0.0263
Coumarins and derivatives	Hydroxycoumarins	8-Hydroxy-7-methoxy-2H-1-benzopyran-2-one	0.0275	0.0011	0.0002	0.0001	0.0001	0.0019
Coumarins and derivatives	Pyranocoumarins	Clausarinol	0.0398	0.0146	0.0229	0.0373	0.0481	0.0516
Flavonoids	Flavones	Apigenin 7-sulfate	1.2303	0.4611	0.2820	0.2189	0.1609	0.1662
Flavonoids	Flavonoid glycosides	Naringenin 4'-O-glucuronide	0.0903	0.0227	0.0060	0.0032	0.0004	0.0007
Flavonoids	Flavonoid glycosides	Pelargonidin 3-sophoroside	0.0718	0.0245	0.0341	0.0478	0.0465	0.0570
Flavonoids	Pyranoflavonoids	Cudraflavone A	0.1037	0.1381	0.1341	0.1408	0.2273	0.2101
Linear 1,3-diarylpropanoids	Chalcones and dihydrochalcones	Dihydronaringenin-O-sulphate	0.4938	0.1170	0.0348	0.0411	0.0116	0.0108
Phenylpropanoic acids	Unclassified	Thiophene-4,5-epoxide	0.3782	0.3205	0.0764	0.1716	0.0317	0.0415
Unclassified	Unclassified	alpha-Tubulin	0.0540	0.1170	0.0761	0.0622	0.0544	0.0512
Unclassified	Unclassified	2,7(14)-Illudadiene-10,15-diol	0.0038	0.0863	0.0263	0.0073	0.0050	0.0042

Note: Group difference were analyzed by ANONA. Metabolites that highly significantly enhanced ($P < 0.01$) were colored red and significantly enhanced ($0.01 < P < 0.05$) were colored pink, while metabolites highly significantly decreased ($P < 0.01$) colored blue and significantly decreased ($0.01 < P < 0.05$) were colored wathet, compared to the non-fasting group.
